# Microcephaly and Zika virus: neonatal neuroradiological aspects

**DOI:** 10.1007/s00381-016-3074-6

**Published:** 2016-04-14

**Authors:** Sergio Cavalheiro, Amanda Lopez, Suzana Serra, Arthur Da Cunha, Marcos Devanir S. da Costa, Antonio Moron, Henrique M. Lederman

**Affiliations:** Department of Neurosurgery, Federal University of Sao Paulo, Rua Botucatu, 591, conj 41, Sao Paulo, SP 04023-062 Brazil; Neurosurgery Service of Hospital da Restauração, Recife, Pernambuco Brazil; Department of Obstetrics, Federal University of Sao Paulo, Sao Paulo, SP Brazil; Department of Diagnostic Imaging, Federal University of Sao Paulo, Sao Paulo, SP Brazil

**Keywords:** Zika virus, Microcephaly, Ventriculomegaly, Lissencephaly, Intracranial calcifications, *Aedes aegypti*

## Abstract

**Purpose:**

The aim of this study is to describe some radiological features in the newborns with microcephaly caused by Zika virus infection during pregnancy.

**Methods:**

We radiologically analyzed 13 cases of newborns with microcephaly born to mothers who were infected by the Zika virus in the early stage of pregnancy.

**Results:**

The most frequently observed radiological findings were microcephaly and decreased brain parenchymal volume associated with lissencephaly, ventriculomegaly secondary to the lack of brain tissue (not hypertensive), and coarse and anarchic calcifications mainly involving the subcortical cortical transition, and the basal ganglia.

**Conclusions:**

Although it cannot be concluded that there is a definitive pathognomonic radiographic pattern of microcephaly caused by Zika virus, gross calcifications and anarchic distribution involving the subcortical cortical transition and the basal ganglia, in association with lissencephaly and in the absence of hypertensive ventriculomegaly, are characteristic of this type of infection.

## Introduction

Latin America is undergoing a major epidemic of Zika virus, which is an arbovirus [1] of the virus family *Flaviviridae* and the genus *Flavivirus*, that is transmitted by Aedes mosquitoes, such as *Aedes aegypti*. In Brazil, it is believed that 1.5 million people were infected in the period between April 2015 and January 2016, whereas Colombia had 13,800 cases. The viral disease is transmitted mainly by the *A. aegypti* mosquito, which has been affecting humans for over 5000 years [1].

The name Zika comes from the Zika forest of Uganda, where the virus was first isolated in 1947 [2], but the Zika infection in humans was described either in Uganda in 1964 and later in Southeast Asia. It is estimated that more than 80 % of infected individuals are asymptomatic [3, 4]. As of January 2016, the virus has been identified in 28 countries and territories. Although the vast majority of cases have occurred after the afflicted individual was bitten by an infected mosquito, some cases have been described to result from sexual transmission from other infected individuals. After the first reports of Zika symptoms (skin rash, fever, arthralgia, headache, and conjunctivitis) were reported, a subsequent increase was reported in cases of Guillain-Barre syndrome and microcephaly [5, 6]. After Zika virus emerged in Brazil, a 20-fold annual increase in the number of microcephaly cases was observed [6, 7]. Identification of the virus in the amniotic fluid, the brain of the newborn, and the placenta, as well as in pregnant women who showed symptoms of infection, strongly indicates that the viral infection destroys the developing brain, consequently resulting in microcephaly [8]. In Brazil, 3670 cases of suspected microcephaly have been tentatively associated with Zika virus, with 404 confirmed cases. The states of northeastern Brazil have been the most affected, especially Pernambuco [9]. The purpose of this study is to evaluate the postnatal neuroradiological findings in 13 patients who were born with microcephaly from mothers who during pregnancy presented signs and symptoms of Zika virus infection.

## Methods

We retrospectively reviewed brain computed tomography and/or magnetic resonance imaging of 13 microcephalic term newborns (head circumference <32 cm). All mothers of these patients showed symptoms of rash during pregnancy. Eleven patients were from Pernambuco, one from Maranhão, and one from Rio Grande do Norte. In 12 cases, the infection symptoms were observed in the first trimester of pregnancy, and the symptoms occurred at the beginning of the fourth month in only one patient. All the patients were born after the 37th gestational week by cesarean section, and all of them had been tested negative for toxoplasmosis, rubella, cytomegalovirus, herpes virus, and syphilis.

## Results

**Fig. 1:**
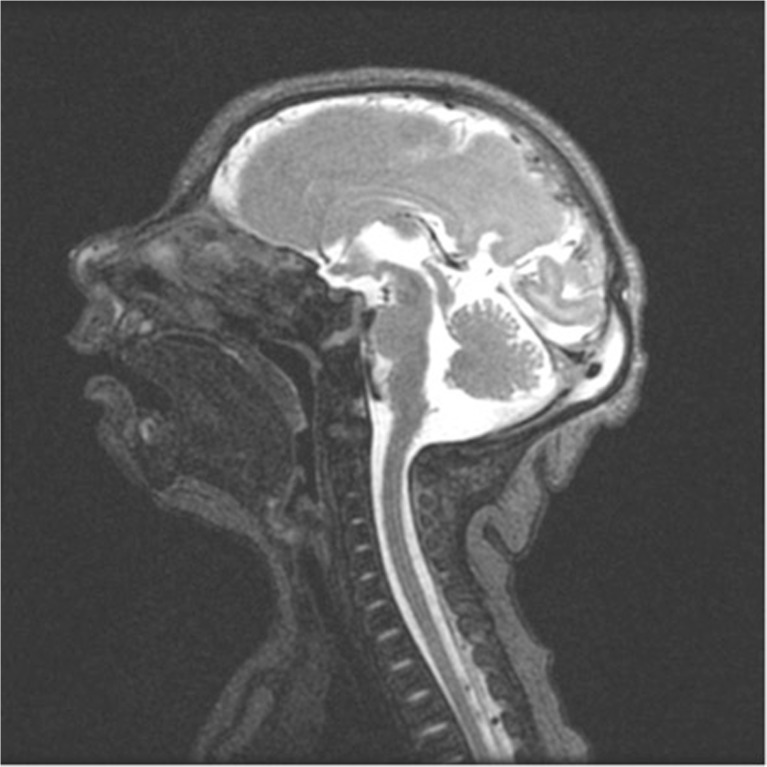
Skull MRI with craniofacial disproportion, increased subarachnoid space, and corpus callosum hypoplasia. The brainstem, cerebellum, and spinal cord are preserved

**Fig. 2:**
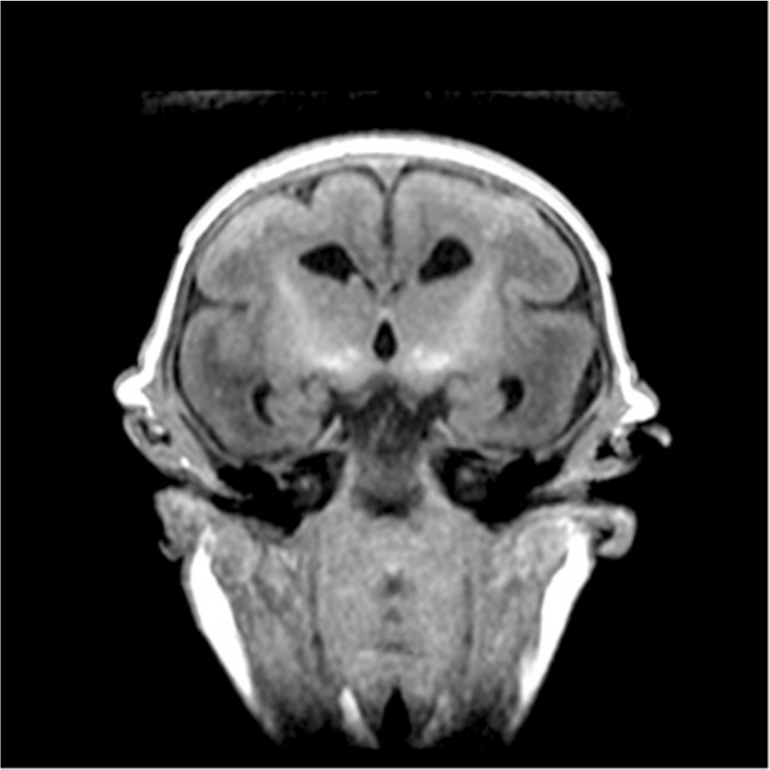
Skull MRI shows telescoped skull, lissencephaly, hypoplasia of the corpus callosum, and calcifications of the basal ganglia

**Fig. 3:**
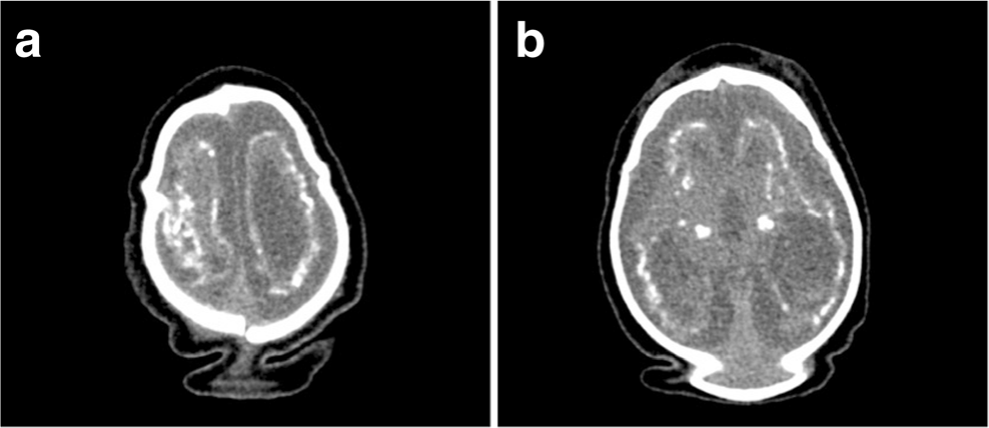
**a** Overriding of the cranial bones, cerebral atrophy, and anarchic distributed coarse calcifications; the excess skin as a result of the decrease in skull volume. **b** CT scan with gross calcifications in the basal ganglia and subcortical cortical transition

**Fig. 4:**
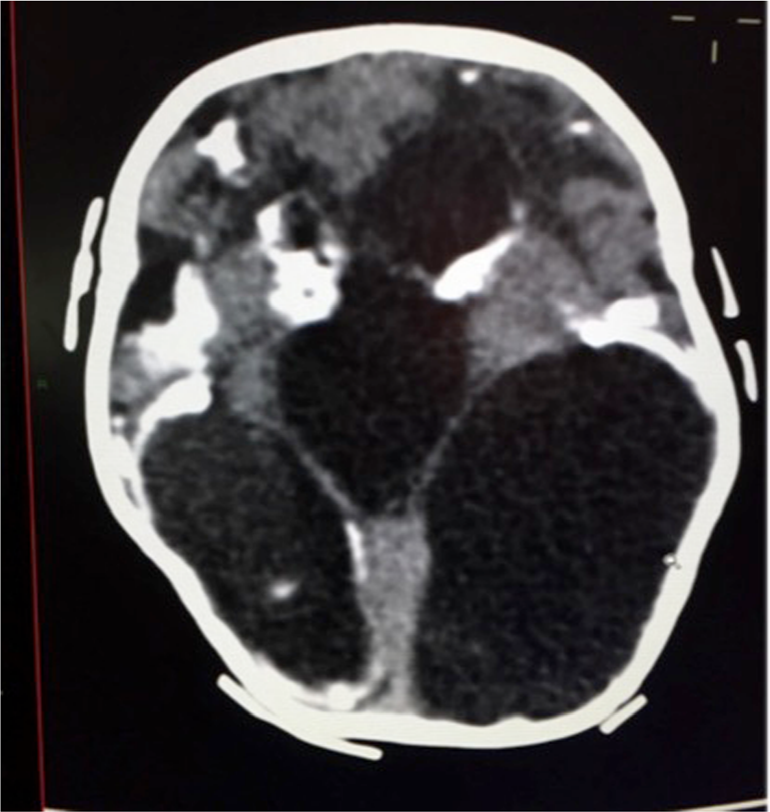
Hypertensive ventriculomegaly with gross parenchymal and basal ganglia calcifications

The following radiological findings were obtained: all patients had craniofacial disproportion with microcephaly (Fig. 1) and a head circumference <32 cm. The skulls had a telescoped aspect with overriding of the bones mainly in the frontal and occipital regions (Fig. 2). The anterior cranial portion showed a diamond shape. Significantly decreased cortical mantle and white matter together with hypoplasia of the corpus callosum was observed in all cases (Fig. 3a). Lissencephaly was observed in all the cases. The subarachnoid space was increased in all cases. Intracranial calcifications were gross and preferably located in the subcortical cortical transition and in the basal ganglia (Fig. 3b). Only one case showed periventricular calcifications. Ventriculomegaly was present in all cases and showed a non-hypertensive aspect in 12 cases, secondary to the loss of the brain. In one case, the lateral ventricles were large and a round third ventricle was seen, demonstrating a hypertensive pattern (Fig. 4). The choroid plexus was large in eight of the 13 patients. In five cases, we noted the presence of intraventricular septations. None of the cases showed schizencephaly. The brainstem, cerebellum, and spinal cord were normal. All of the neuroradiological aspects are described in Table 1.

**Table 1:** Neuroradiological findings in 13 cases of microcephaly, probably secondary to the Zika virus infection

Radiological features	Number of cases (13)
Craniofacial disproportion (microcephaly)	13
Skull telescoped with overriding of bones	13
Decreased cerebral mantle	13
Increased subarachnoid space	13
Lissencephaly	13
Ventriculomegaly	13
Hypoplasia of the corpus callosum	13
Coarse calcifications involving the subcortical cortical transition and the basal ganglia	12
Large plexus choroid	8
Intraventricular septations	5
Periventricular calcifications	1
Brain stem atrophy	0
Cerebellar atrophy	0
Ependymitis	0
Spinal atrophy	0
Schizencephaly	0

## Discussion

Several viruses can affect the fetus during the embryonic period, causing brain damage. Many of these injuries are inflammatory and characterized by ependymitis and small intraventricular hemorrhage with secondary hydrocephalus, or they cause real destruction of the brain parenchyma with cell growth arrest, apoptosis, calcifications, cerebral malformations, and microcephaly. Infection earlier in gestation results in the most severe damage to the brain parenchyma, between 8 and 12 weeks (i.e., during the organogenesis of the brain), structural abnormalities can develop. One of the first associations between maternal infection and fetal disease was attributed to Morquio [10], who reported cases of hydrocephalus in children of mothers with smallpox in 1903. In Brazil in 1964, Machado de Almeida [11] demonstrated a relationship between maternal smallpox and the presence of fetal hydrocephalus when maternal symptoms occurred in the last two trimesters of pregnancy. In two cases where necropsy was performed, periventricular calcifications were observed that did not appear on x-rays, and ependymitis without the presence of brain malformations was also observed. Swan et al. in 1943 [12] and Swan in 1944 [13] concluded that when a woman contracts rubella in the first 2 months of pregnancy, the likelihood of giving birth to a child with congenital defects is 100 %, whereas the risk is reduced to 50 % if the disease focus is in the third month. The microcephaly resulting from rubella is explained by chronic leptomeningitis and secondary vascular lesions. Barkovich and Lindan in 1994 [14] reported cytomegalovirus infection results in inadequate perfusion of the placenta that results in ischemia and that the virus has special affinity for immature cells of the germinal matrix that would result in loss of brain tissue and abnormalities of the cortex. Early infection with cytomegalovirus between 16 and 18 weeks of gestation, i.e., occurring at the onset of the neuronal migration, can lead to lissencephaly.

Recently, Mlakar et al. (2016) [8] demonstrated some evidence of Zika virus neurotropism, by isolating viral particles solely in the neural tissue of a fetus aborted with 37 weeks of gestation and whose mother presented signs of Zika infection in the 13th week of gestation. Moreover, Abidi et al. (2016) [15] suggested two hypotheses to explain the mechanisms in which the Zika virus infection causes the microcephaly in pregnant women. The first hypothesis considers the possibility of direct delivery of virus through the placenta and his neurotropism causing brain tissue damage in an early stage of the pregnancy. The second considers the placenta mediates a response to Zika virus infection that could change the profile of inflammatory markers in the fetal tissues.

The radiological aspects of Zika virus infection identified in the present study suggest acute destruction of the brain parenchyma, with evidence of cell migration abnormalities leading to intracranial hypotension, followed by overriding of the skull bones, filling of the space between the brain and bone with cerebrospinal fluid, and microcephaly. The shape of the skull in the anterior portion closely resembles the so-called lemon sign found in fetuses with intracranial hypotension in Chiari malformation type II with myelomeningocele and cerebrospinal fluid leakage. In contrast to previous observations for other viruses, we found an anarchic pattern of intracranial calcifications, with gross calcifications, involving the subcortical cortical transition and the basal ganglia. Periventricular calcification was found in only one case. The location of calcifications suggests that the destruction of the brain parenchyma occurs by vasculopathy and not by ependymitis or bleeding as seen in cases of coxsackie infection. We also observed preservation of the brainstem, the cerebellum, and the medulla, suggesting that viral involvement spared the vertebrobasilar circulation, mainly the carotid system. The presence of lissencephaly suggests that in most cases the infection occurred earlier than 18 weeks of gestation. Viral tropism to the germinal matrix and apoptosis may also explain these findings. We did not observe cases of schizencephaly, similarly to the findings of Schuler-Faccini et al. [7] in 35 patients. The viral pathophysiology of this type of infection and the type of immunity obtained following the primary infection remain unclear. If the immunity is similar to that against the rubella virus, vaccination may be able to eradicate the fetal disease. However, if immunity is not complete, a second exposure to the virus during pregnancy would not prevent viremia, and therefore, damage to the fetal nervous system may occur.

## Conclusion

Although it cannot be concluded that there is a definitive pathognomonic radiographic pattern of microcephaly caused by Zika virus, coarse calcifications and anarchic distribution involving the subcortical cortical transition and the basal ganglia, in association with lissencephaly and in the absence of hypertensive ventriculomegaly, are characteristic of this type of infection. We also believe that during the maternal infection, viremia with vasculitis in the carotid brain circulation and brain tissue necrosis occurs, with evidence of cell migration abnormalities. The coarse severe calcifications are considered to be part of the dysmorphic calcification from the healing phase.

A larger study with more cases is needed to prove that these findings could contribute to the radiological diagnosis of this aggressive pandemic viral disease.
